# Corrosion behavior of silver-coated conductive yarn

**DOI:** 10.3389/fchem.2023.1090648

**Published:** 2023-03-22

**Authors:** Jihye Park, Sun Hwa Park, Soo-Hwan Jeong, Jung-Yong Lee, Jae Yong Song

**Affiliations:** ^1^ Interdisciplinary Materials Measurement Institute, Korea Research Institute of Standards and Science, Daejeon, Republic of Korea; ^2^ Department of Electrical Engineering, Korea Advanced Institute of Science and Technology, Daejeon, Republic of Korea; ^3^ Department of Chemical Engineering, Kyungpook National University, Daegu, Republic of Korea; ^4^ Department of Semiconductor Engineering, Pohang National University of Science and Technology, Pohang, Republic of Korea

**Keywords:** conductive yarn, silver corrosion, sodium chloride, galvanic corrosion, partcoating of gold

## Abstract

The corrosion mechanism and kinetics of the silver-coated conductive yarn (SCCY) used for wearable electronics were investigated under a NaCl solution, a main component of sweat. The corrosion occurs according to the mechanism in which silver reacts with chlorine ions to partly form sliver chloride on the surface of the SCCY and then the local silver chloride is detached into the electrolyte, leading to the electrical disconnect of the silver coating. Thus, the electrical conductance of the SCCY goes to zero after 2.7 h. The radial part-coating of gold, which is continuously electrodeposited in the longitudinal direction on the SCCY but is partly electrodeposited in the radial direction, extends the electrical conducting lifetime up to 192 h, despite the corrosion rate increasing from 129 to 196 mpy (mils per year). Results show that the gold partly-coating on the SCCY provides a current path for electrical conduction along the longitudinal direction until all the silver underneath the gold coating is detached from the SCCY strands, which creates the electrical disconnect. Based on the corrosion behavior, i.e., local oxidation and detachment of silver from the SCCY, the gold part-coating is more cost effective than the gold full-coating electrodeposited on the entire surface for electrically conducting SCCY.

## 1 Introduction

Textile-based wearable electronics, which are composed of biosensors, semiconducting chips, and energy storage devices, have been extensively investigated to monitor the vital signals of the human body (Service, 2003; Müller et al., 2011; Stoppa and Chiolerio, 2014; Wen et al., 2016; Huang et al., 2017; Heo et al., 2018). Those electronic devices are generally interconnected by conductive yarns that are embedded into a textile (Linz et al., 2006). Occasionally, conductive yarns are used as a sensor electrode by sewing on fabrics because soft, comfortable, and wearable textiles are still preferred rather than rigid devices (Post et al., 2000; Paradiso et al., 2004; Stoppa and Chiolerio, 2014).

The conductive yarn might be a bundle of twisted polymer fibers coated with metals or a mixture of non-conductive fibers and metal wires such as silver, copper, platinum, stainless steel and gold (Xu et al., 2008; Beckmann et al., 2010; Stoppa and Chiolerio, 2014; Liu et al., 2018). Although copper is a representative material for metal lines due to its facile synthesis and low-cost, it tends to be easily oxidized in air, leading to high electrical resistance at surface (Leygraf, 2011). And stainless steel, one of the metal line candidates due to its anti-corrosion feature, is costly owing to the complicate production process (OSHIMA et al., 2007). Comparatively, silver has attracted much attention due to its high electrical conductivity, easy synthesis process, and less corrosion (Liu et al., 2018). Thus, silver is widely used for interconnection in the application fields such as wearable sensors and smart textiles, which require the flexible electrical routing on flexible substrate (Stoppa and Chiolerio, 2014). However, silver is also vulnerable to oxidation in the ambient atmosphere (Rooij, 1989) and its corrosion is accelerated by atmospheric contaminants and humidity (Mcneil and Little, 1992; Salas et al., 2012; Lin et al., 2013). Furthermore, in a chloride-containing environment, e.g., sweat, silver is readily oxidized and results in the predominant formation of silver chloride (Graedel, 1992; Ha and Payer, 2011; Salas et al., 2012). Thus, silver-coated conductive yarn (SCCY) tends to be easily corroded by sweat, which is composed of 99% water and a main solute of sodium chloride, when it is used in textile-based wearable electronics. The corrosion of SCCY degrades the performance and lifetime of wearable electronics due to short circuits and electrical interference (Graedel, 1992; Zhang and Tao, 2005; Ha and Payer, 2011). Recently, it was reported that the physical and chemical state of the human body, such as in exercise and emotional arousal, can modify significantly the electrolyte concentration of sweat and accelerate the corrosion process (Fukumoto et al., 1988; Schazmann et al., 2010; Dooren et al., 2012; Nyein et al., 2016). Until now, the corrosion mechanism and corrosion rate of silver conductive yarns has not been clear, in the viewpoint of the chlorine concentration in the electrolyte.

In this work, we investigated the corrosion mechanism of commercial SCCY with regard to the concentration and temperature of the NaCl solution and evaluated its electrical lifetime. We introduced gold part-coating to extend the electrical lifetime of the SCCY and analyzed its roles for practical applications. Gold, which might be easily electrodeposited, was selected due to its chemical stability in NaCl solution and good adhesion through metallic bonding with silver.

## 2 Materials and methods

### 2.1 Electrochemical tests

The silver-coated conductive yarn (Swicofil, China) was dipped into a buffered oxide etchant solution (J.T.Baker, United States) for 60 s, rinsed in distilled water, and dried. Electrochemical measurements were carried out using a three-electrode system (Modulab 2100A, Solartron). A coiled Pt wire (0.5 mm in diameter and 1 m in length) and a KCl saturated Ag/AgCl electrode were used as counter electrode and reference electrode, respectively. Three strands of SCCY with an exposed length of 2 cm as a working electrode were attached to a glass substrate using epoxy resin. The potentio-dynamic polarization measurements were conducted in the range of open circuit potential (OCP, ±100 mV) at a scan rate of 1 mV/s. All the measurements were repeated four times under various conditions: in electrolyte concentrations of 0.01, 0.1 and 1 M NaCl (Guaranteed reagent, Junsei, Japan) and at temperatures of 30, 40, 50 and 60°C. An immersion test was carried out in a 1 M NaCl solution at 60°C for 3.5 h without agitation, where two SCCY strands with an exposed length of 1 cm were used as a working electrode.

### 2.2 Morphology and microstructure characterization

The morphology of the SCCY was analyzed by a field-emission scanning electron microscope (SEM, Hitachi S-4800). The cross-sectional SEM image of the SCCY was obtained after the focused ion beam etching process (FIB, Helios Nanolab 450F1, FEI). The crystal structure and chemical composition of the SCCY were analyzed by an X-ray diffraction instrument (XRD, SmartLab X-ray Diffractometer, Rigaku) and an energy dispersive X-ray spectroscopy detector (EDS, EDAX Genesis XM4, Bruker). The apparent electrical conductance of the SCCY was measured for the interval of 1 cm using the electrical multimeter (FLUKE 115).

### 2.3 Partly gold-coated SCCY

SCCY with an exposed length of 3 cm, which was attached to a glass substrate using epoxy resin, was immersed in ethanol for 3 min in order to improve water wettability on the surface and then it was rinsed in distilled water. The gold layer was electrochemically deposited on SCCY using a three-electrode system in 100 μM HAuCl_4_·H_2_O (n = 3.5, Kojima Chemicals Co., #903060) with an external bias voltage of −0.3 V for 5 min. The gold was electrodeposited on the silver surface exposed to the electrolyte. Thus, the gold was partly electrodeposited in the radial direction of the strand, but was continuously deposited in the longitudinal direction. The thickness of the partly gold-coating layer was estimated to be approximately 32 nm using the EDS analysis of the atomic concentration ratio (92–8) of the Ag and Au elements.

## 3 Results and discussion

### 3.1 Surface morphology and microstructure of SCCY

**FIGURE 1:**
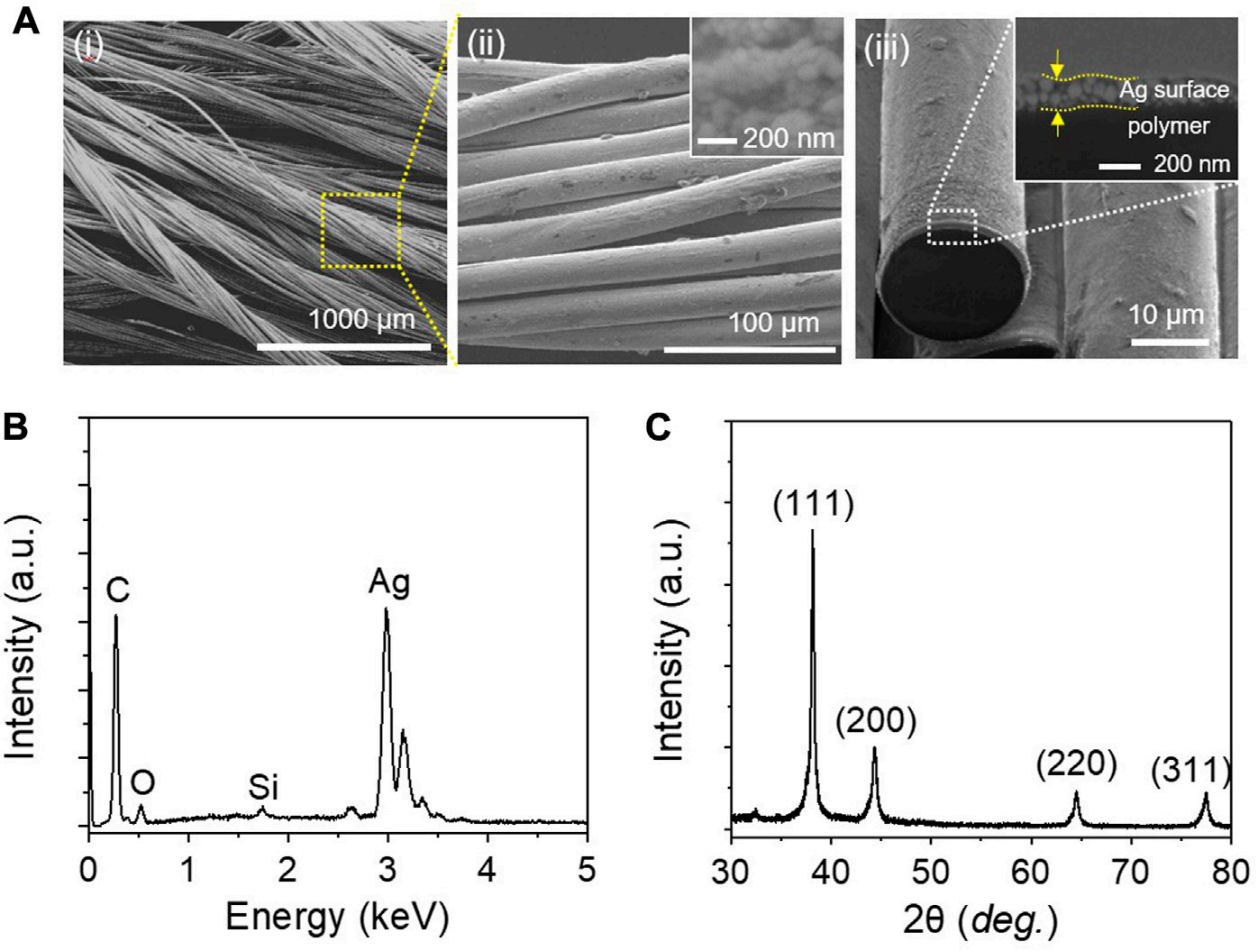
**(A)** Typical SEM images of SCCY: (i) multifilament yarn, (ii) single filament (inset: top view of the silver surface), (iii) single strand (inset: enlarged cross-sectional SEM image of silver layer marked as the white dashed area, **(B)** EDS spectra and **(C)** XRD pattern of SCCY.

Figures 1Ai shows a typical SEM image of SCCY that is composed of many filaments with a twisted structure of strands. Figures 1Aii is the magnified SEM image and the filament marked by the yellow box in i is composed of twisted strands (Mouthuy et al., 2015). A strand has a diameter of 20 μm, and its entire surface is densely coated with a uniform silver layer composed of small grains, as shown in the insets of Figures 1Aii, iii. With the help of the FIB etching process, the cross-sectional SEM image shows that the granular silver layer had a thickness of approximately 185 nm (Figures 1Aiii). In Figure 1B, the EDS spectra indicate that the chemical elements of silver and carbon come from the conductive silver layer and non-conductive polymer fiber, respectively. In Figure 1C, all the XRD peaks reflect the crystallographic planes (111), (200), (220) and (311) of silver, indicating a face-centered cubic crystal structure (JCPDS # 087-0718).

### 3.2 Corrosion rate of SCCY

**FIGURE 2:**
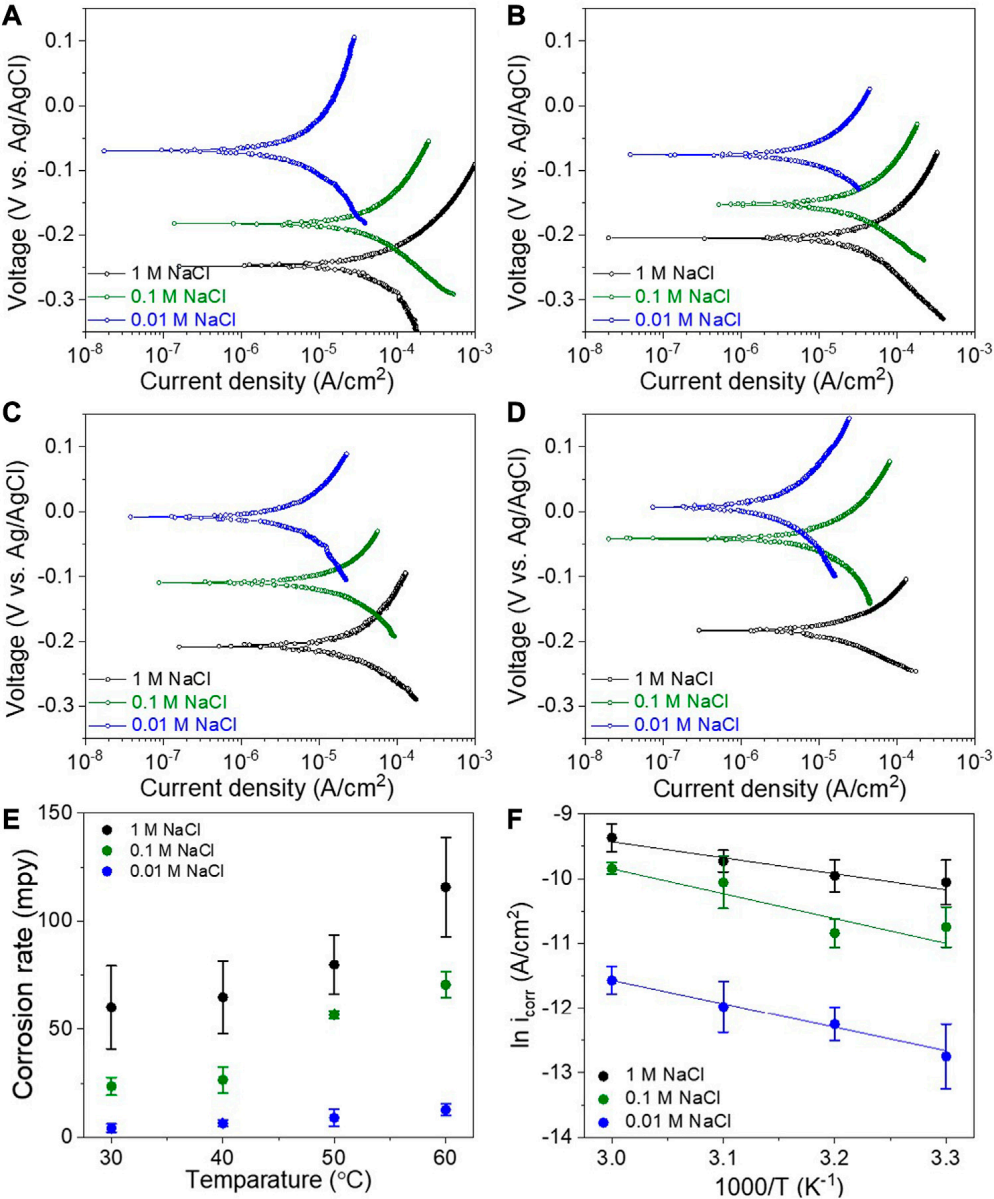
Potentiodynamic polarization curves of SCCY in various concentrations of 1, 0.1, and 0.01 M NaCl solution at **(A)** 60°C, **(B)** 50°C, **(C)** 40°C, and **(D)** 30°C, **(E)** variation of the corrosion rate with the NaCl concentration and temperature of the electrolyte, and **(F)** Arrhenius plots of ln icorr depending on 1000/T with the NaCl concentration.

Figure 2 shows the variations of the potentio-dynamic polarization behavior with the temperature (from 30 to 60°C) and concentration (from 0.01 to 1 M) of the NaCl electrolyte. The electrochemical parameters of the corrosion potential (*E*_corr_) and corrosion current density (*i*_corr_) were determined from the points intersecting between the anodic branch and cathodic branch, where the anodic and cathodic reaction rates were equivalent, as summarized in Table 1 (Stern and Geary, 1957; Power and Ritechie, 1987; Jones, 1992). According to Faraday’s law, the corrosion rate can be evaluated by the Tafel extrapolation method for the potentio-dynamic polarization curve, as follows (Jones. 1992; Cao et al., 2019):

$$Corrosion\;rate=\left(i_{corr}\times M\right)/\;\left(n\times F\times \rho \right)$$ (1)

where *M* is the atomic weight of silver (107.8 g), *n* is the number of electrons involved in the reaction (*n* = 1), *F* is Faraday’s constant (96,500 C/mol), and *ρ* is the density of silver (10.49 g/cm^3^).

**TABLE 1:** Electrochemical parameters obtained from the potentiodynamic polarization curves in Figure 2.

Electrolyte		Electrochemical parameters	
Temperature (°C)	Concentration (M)	*E*_*corr*_ (mV vs. Ag/AgCl)	*i*_*corr*_ (µA/cm^2^)
60	1	−266.8	97.3
	0.1	−191.9	55.0
	0.01	−80.6	10.0
50	1	−220.5	58.8
	0.1	−163.3	42.0
	0.01	−77.9	8.5
40	1	−207.4	37.8
	0.1	−112.0	18.7
	0.01	0	6.2
30	1	−189.4	23.8
	0.1	−43.8	15.3
	0.01	13.1	4.4

Figure 2A shows the potentio-dynamic polarization curves with concentrations of 0.01, 0.1 and 1 M at 60°C. With an increase in electrolyte concentration, the *E*_corr_ shifted in the negative direction from −80.6 to −266.8 mV, indicating more activation of the anodic reaction (Power and Ritechie, 1987; Cao et al., 2019). In addition, the *i*_corr_ increased from 10.0 to 97.3 μA/cm^2^ and consequently, the corrosion rate of the SCCY increased from 13 to 129 mpy (mils per year), as shown in Figure 2E. It is supposed that the silver layer of the SCCY is so sensitive as to react with the chlorine ions in the NaCl electrolyte, and its corrosion is controlled by Cl ion transfer from the electrolyte to the silver surface of the SCCY (Graedel, 1992; Mcneil and Little, 1992; Ha and Payer, 2011; Lin et al., 2013). In a similar vein, Figures 2B–D respectively show that each corrosion potential and corrosion current density increased with an increase in the NaCl concentration at all the temperatures. Figure 2E shows the SCCY corrosion rates varying with the temperature and NaCl concentration of the electrolyte. It is noted that the corrosion rate was strongly correlated with the increasing temperature and NaCl concentration. When the electrolyte temperature increased from 30 to 60°C in a 1 M NaCl solution, the *i*_*corr*_ increased from 23.8 to 97.3 μA/cm^2^ and the *E*_*corr*_ moved toward the active anodic reaction from −189.4 to −266.8 mV, as shown in Table 1. As a result, the corrosion rate of the SCCY increased from 32 to 129 mpy with the temperature increasing from 30 to 60°C for the 1 M solution. Similar behaviors of the corrosion rate varying with the increase of the electrolyte temperature were also observed for 0.01 and 0.1 M NaCl solutions. The corrosion rate can be described as a function of the electrolyte temperature, following the Arrhenius equation (Jones, 1992; Blasco-Tamarit et al., 2008):

$$\ln\left(i_{corr}\right)=\ln i-E_{a}/RT$$ (2)

where *E*_a_ is the activation energy of the corrosion process, *R* is the gas constant (8.314 J/molK), *T* is the electrolyte temperature, and *i* is the current density. Figure 2F shows the linear relationship between ln *i*_corr_ and 1/*T*, resulting in the *E*_a_ of 19 kJ/mol for SCCY corrosion in a 1 M NaCl solution. The activation energy increased to 29 kJ/mol and 32 kJ/mol for the NaCl concentrations of 0.1 and 0.01 M, respectively. This indicates that the energy barrier for the SCCY corrosion reaction *E*_a_ decreased with an increase of the chlorine concentration in the electrolyte. Thus, the concentration and temperature of the NaCl solution play the role of initiating and accelerating the corrosion reaction of the SCCY.

### 3.3 Corrosion behavior of SCCY

**FIGURE 3:**
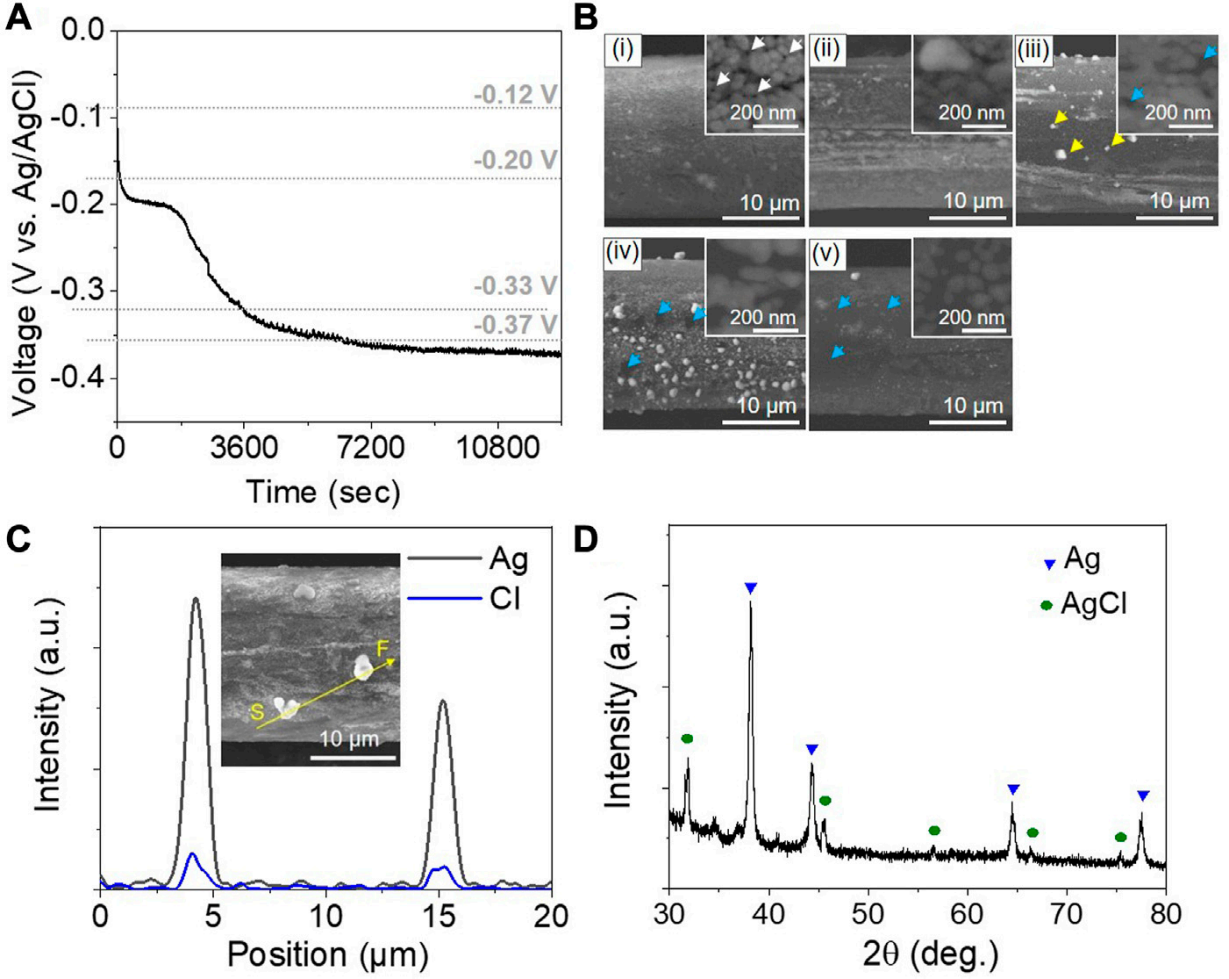
**(A)** Variation of open circuit potential (OCP) with the immersion time in a 1 M NaCl solution at 60°C. **(B)** SEM images of a strand of SCCY with the immersion times of (i) 200, (ii) 2000, (iii) 4000, (iv) 10000, and (v) 15000 s. The insets indicate magnified SEM tip-view images. (White, yellow, and blue arrows indicate pinholes, silver chloride particles, and polymer fiber, respectively.) **(C)** EDS line spectra of silver and chlorine on a strand of SCCY after an immersion time of 4000 s in a 1 M NaCl solution. **(D)** XRD pattern of the SCCY after an immersion time of 10,000 s in a 1 M NaCl solution at 60 °C.

Figure 3A shows the variation of the open circuit potential (OCP) with the immersion time of the SCCY in a 1 M NaCl solution at 60°C. The initial OCP of −0.12 V abruptly reached −0.20 V in the negative direction. At the stage of the OCP drop, many pinholes were observed on the surface of the SCCY, as indicated by the white arrows in Figures 3Bi. They were located at the grain boundaries which were energetically more activated for silver atoms to be easily dissolved into the electrolyte (Beaunier, 1982; Blasco-Tamarit et al., 2008). When the OCP decreased to −0.2 V for 2,000 s, the SCCY surface became rough with a distinguished granular structure (Figures 3Bii). With a further decrease of the OCP from −0.2 to −0.33 V, μm-sized particles appeared, as marked by the yellow arrows in iii. According to the EDS analyses, the particles were composed of silver and chlorine elements (Figure 3C). The OCP eventually approached −0.37 V and remained constant with a further immersion time up to 15,000 s. It is noted that the number density of the particles on the surface of SCCY increases, as shown in Figures 3Biv. XRD analyses for the sample of Figures 3Biv clearly exhibit that the particles were the AgCl phase with a face-centered cubic crystal structure (JCPDS # 014-0255), as shown in Figure 3D. It is reasonable for the AgCl phase to form spontaneously through the corrosion process of SCCY in a NaCl solution, since the Gibbs free energy (∆G) for the formation of AgCl is known to be −110 kJ/mol in this environment (Thompson et al., 2000; Wan et al., 2012; Wan et al., 2015; Pargar et al., 2017). The corrosion reaction can be assumed to occur, as follows (Graedel, 1992; Ha and Payer, 2011):

$$Ag\left(s\right)+{Cl}^{-}\left(aq\right)\;\to \;AgCl\left(s\right)+e^{-}$$ (3)

According to the corrosion reaction of Eq. 3, silver is consumed to form the silver chloride on the surface of SCCY, as shown in Figures 3Bi–iv. It is presumed that the silver chloride initially nucleated at grain boundaries, resulting in the formation of the pinholes, and then grew to form large particles *via* the Ostwald ripening mechanism near the interface between silver and the electrolyte (Roosen and Carter, 1998; Park et al., 2014). Finally, on complete consumption of silver on the local surface of SCCY, the AgCl particles were detached from the polymer surface exposed to the electrolyte, as indicated by the blue arrows of Figures 3Biv, v. In this way, the corrosion of silver led to the electrical disconnect of the SCCY. Here, two possibilities are that the silver chloride particles were physically detached from the surface and that the chloro-complexes (AgCl_x_^−(x−1)^) of silver progressively formed to be dissolved in chloride media (Dinardo and Dutrizac, 1985; Roosen and Carter, 1998; Li et al., 2010; Levard et al., 2013). Since the ∆G of the ionic silver compound is negative, the chloro-complexes, such as AgCl_2_^−^ and AgCl_4_^3-^, also formed. It has been reported that the formation of soluble AgCl_x_^−(x−1)^ is strongly dependent on the chloride ion concentration; at low Cl^−^ concentrations, the AgCl solid phase formed on the Ag surface, while at high Cl^−^ concentrations, Ag dissolves faster due to the rapid formation of soluble silver compounds (Dinardo and Dutrizac, 1985; Roosen and Carter, 1998; Li et al., 2010; Levard et al., 2013).

### 3.4 Electrical conducting lifetime of SCCY

**FIGURE 4:**
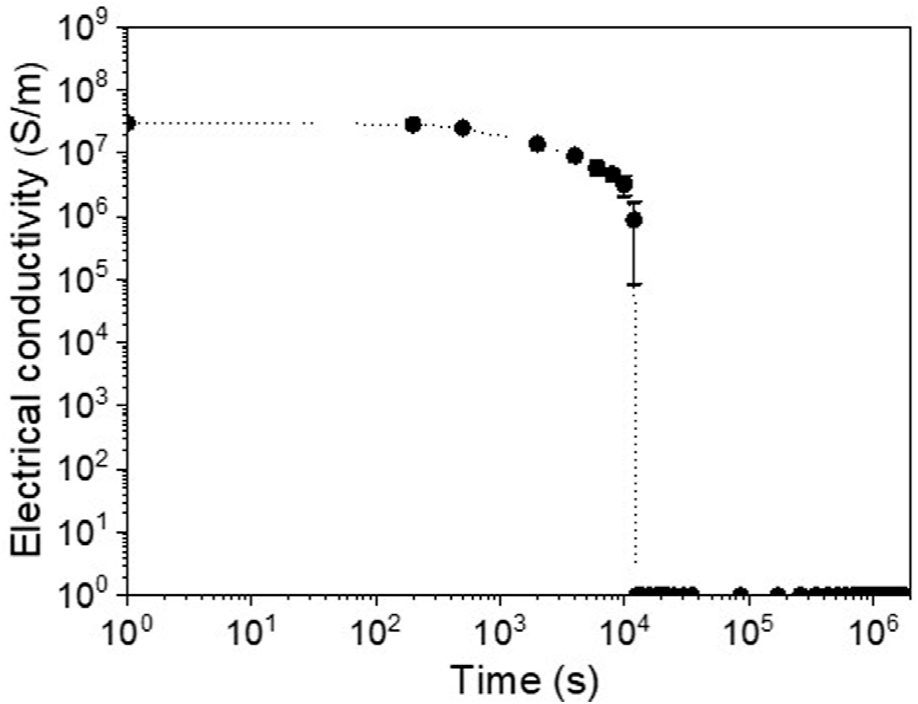
Electrical conductivity of the SCCY as a function of immersion time in a 1 M NaCl solution at 60°C.

The electrical conductivity of the SCCY depended upon the change of its surface morphology under a chloride-containing solution. Figure 4 shows the variation of the electrical conductivity of the SCCY with the immersion time in a 1 M NaCl solution at 60°C. The initial conductivity of about 3 × 10^7^ S/m remained constant and then slowly decreased near 2,000 s, and abruptly went to null after 10,000 s. This agreed well with the OCP behavior in which the OCP reached near −0.37 V after 10,000 s (Figure 3A). Therefore, the deterioration of the electrical conductivity was due to the corrosion mechanism of silver following Eq. 3. Using the retention time of the apparent electrical conductivity shown in Figure 4, we evaluated the corrosion rate of the 185 nm-thick silver layer to be approximately 67 nm/hr, which was much lower than that (370 nm/hr, i.e., 129 mpy) evaluated by the potentiometric polarization test shown in Figure 2. The discrepancy might be due to the different method in measuring the electrical conductivity, i.e., for the immersion test, the SCCY can be electrically interconnected by several live strands which serve as a current path and accordingly, the corrosion rate may be estimated much lower.

### 3.5 Corrosion behavior of partly gold-coated SCCY

**FIGURE 5:**
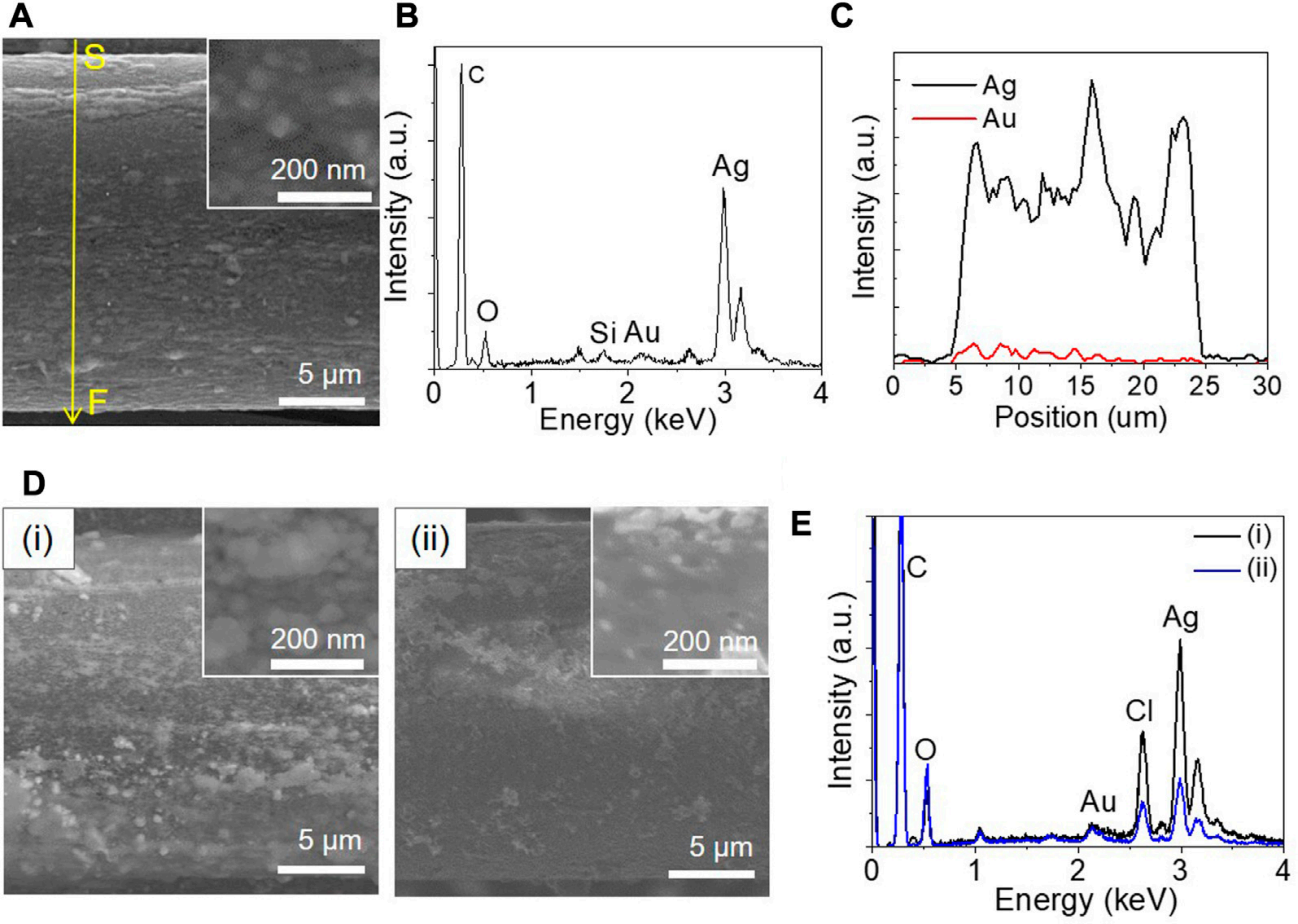
**(A)** SEM image of a partly gold-coated strand (the inset indicates the enlarged top view images of the gold surface). **(B)** EDS spectra and **(C)** compositional variation of silver and gold elements along the scan line from S to F indicated by the yellow arrow in **(A)**. **(D)** SEM images and **(E)** EDS spectra of the partly gold-coated strand after the immersion time in 1 M NaCl solution at 60°C for (i) 35,000 and (ii) 864,000 s, respectively. (The insets indicate the enlarged SEM images of the top view).

In order to extend the electrical lifetime of SCCY, the surface of the SCCY, which might be exposed to the NaCl solution, was partly coated with 8 at% gold, because gold is more robust against chloride-containing solution than silver (Revie, 2011; Park et al., 2018). As silver tends to be easily oxidized in an aqueous gold (III) chloride solution and Au^3+^ ions are likely to be reduced to form a porous structure, the electrodeposition of gold was carried out under an external bias voltage of −0.3 V (Xia et al., 2013). Figure 5A shows a typical SEM image of the gold layer electrodeposited on the Ag surface. According to the EDS analyses shown in Figures 5B,C, the gold layer was partly deposited on the only Ag surface exposed to the electrolyte. Figure 5D shows the time-dependent morphology of the partly gold-coated SCCY which was immersed in a 1 M NaCl solution at 60°C and many AgCl particles were formed after the immersion of 35,000 s Figures 5Di and then disappeared on the surface after 864,000 s Figures 5Dii. According to the EDS analyses shown in Figure 5E, the content of Ag and Cl was significantly decreased for the sample with an immersion time of 864,000 s, while the gold content remained almost constant. This was consistent with the result where the silver was oxidized to form AgCl particles and then disappeared on the surface, as shown in Figures 5Dii.

**FIGURE 6:**
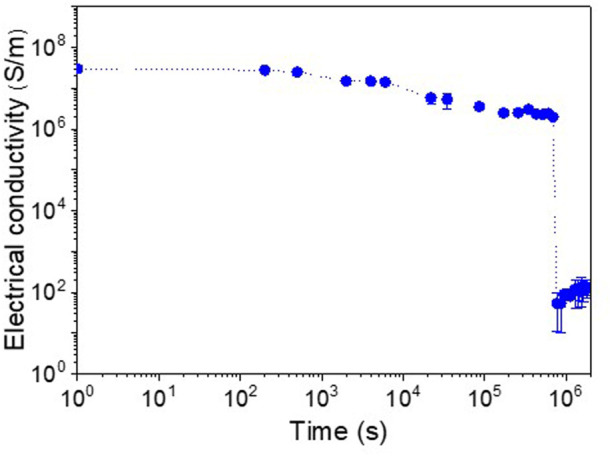
Electrical conductivity of the partly gold-coated SCCY as a function of immersion time in a 1 M NaCl solution at 60°C.

Figure 6 shows the variation of the electrical conductivity with the immersion time for the partly gold-coated SCCY which was immersed in a 1 M NaCl solution at 60 °C. The electrical conductivity (3 × 10^7^ S/m) slowly decreased to 2 × 10^6^ S/m up to 690,000 s and then abruptly decreased to 10^2^ S/m, an insulating level.

**FIGURE 7:**
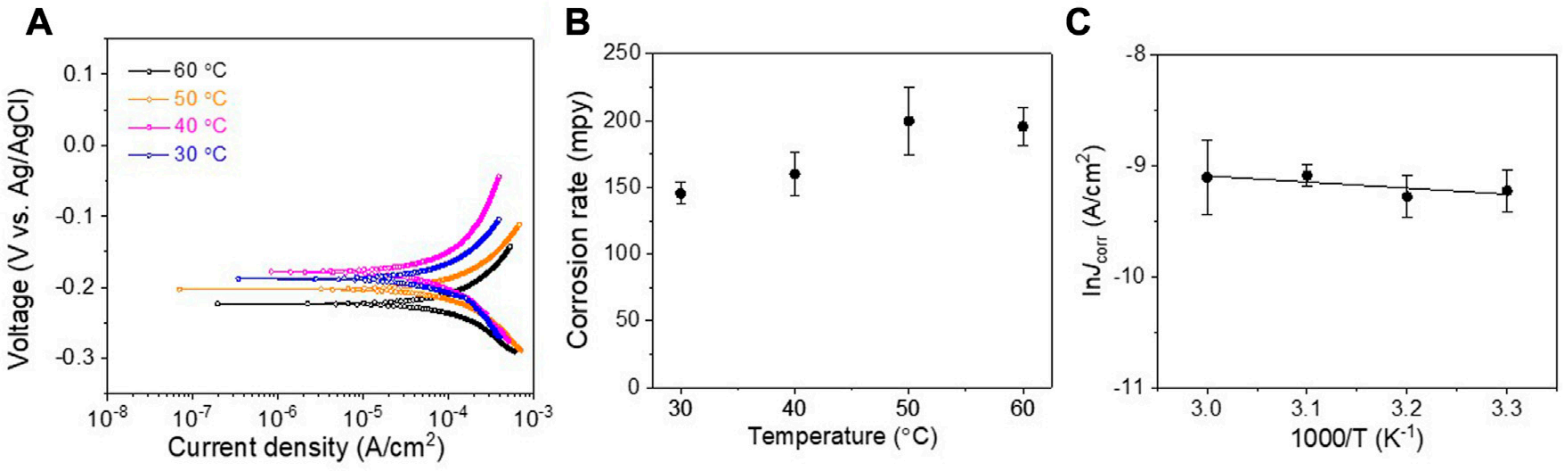
**(A)** Potentio-dynamic polarization curves of the partly gold-coated SCCY in 1 M NaCl solution at 30, 40, 50, and 60°C, respectively. **(B)** The corrosion rate and **(C)** Arrhenius plot of ln *i*_*corr*_ vs. 1000/T for the partly gold-coated SCCY.

**FIGURE 8:**
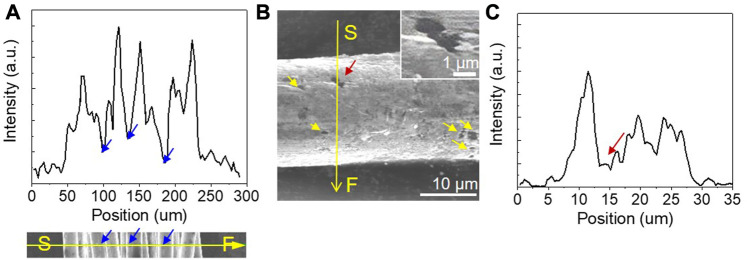
SEM images of **(A)** a filament and **(B)** a strand of the partly gold-coated SCCY (inset: an enlarged image marked by the red arrow). **(A, C)** EDS spectra of gold content variation along the scan line from S to F shown in **(A, B)**, respectively.

To further understand the corrosion kinetics of the gold partly-coated SCCY, the potentio-dynamic polarization analyses were performed with the electrolyte temperatures of 30°C–60°C in a 1 M NaCl solution (Figure 7A). The *E*_*corr*_ of the partly gold-coated SCCY increased with the temperature and reached −229.1 mV at a temperature of 60 °C, which was slightly more positive than that (−266.8 mV) of the SCCY. As shown in Table 2; Figure 7B, the *i*_*corr*_ value and the corrosion rate of the partly gold-coated SCCY at 60°C were evaluated as 148.0 μA/cm^2^ and 196 mpy, respectively, which were much higher than those of the SCCY at 60°C (97.3 μA/cm^2^ and 129 mpy). According to Eq. 2, the activation energy (*E*_*a*_) for corrosion of the partly gold-coated SCCY was 4.6 kJ/mol, as shown in Figure 7C, which was much lower than that for the SCCY (19 kJ/mol), indicating the accelerated oxidation of the gold partly-coated SCCY. The increased corrosion rate for the partly gold-coated SCCY was likely due to the galvanic corrosion in which when two dissimilar metals with different electrode potentials are in contact in the presence of an electrolyte, the more reactive metal transfer electrons to the less reactive one by forming an equilibrium (Fuller and Harb, 2018). Here, as the partly gold-coated region and the uncoated silver are in contact, the silver, which was exposed to the electrolyte, corroded more quickly according to the galvanic corrosion mechanism. Figure 8A shows the EDS line spectra corresponding to the SEM image of the filament that has a non-uniform distribution of gold content in the radial direction. It seems that there was not much gold electrodeposited on the Ag surface which was not exposed to the electrolyte, as marked by the arrows in the EDS spectra corresponding to the SEM image. Each strand had many pits on the surface, as indicated by the arrows in Figure 8B. The pits had little gold deposited on them, as seen by the EDS line spectra in Figure 8C. The pits might act as the path needed for the NaCl solution to reach the Ag surface. Since the silver exposed to the electrolyte through the pits can act as a sacrificial anode due to the galvanic corrosion, the partly gold-coated SCCY showed higher corrosion rates.

**TABLE 2:** Electrochemical parameters estimated from Figure 7A.

NaCl solution	Electrochemical parameters	
Temperature (°C)	*E*_*corr*_ (mV vs. Ag/AgCl)	*i*_*corr*_ (µA/cm2)
60	−229.1	148.0
50	−207.9	151.0
40	−190.5	121.0
30	−186.4	110.1

It is noted that the electrical conducting lifetime of the partly gold-coated SCCY was approximately 690,000 s, which was 69 times higher than that (10,000 s) of the SCCY, despite the increased corrosion rate. This suggests that the partly-coated gold, which does not prevent the silver from corroding, can act as a current path until the silver layer underneath completely dissolved into the electrolyte, although the entire silver surface was not fully coated with gold.

## 4 Conclusion

The corrosion behavior of SCCY in NaCl solution was investigated for application to textile-based wearable devices. The corrosion of silver occurred on the surface of the SCCY as follows: the dissolution of silver layer and the formation and removal of silver chloride in sequence. The corrosion rates increased with an increase in concentration and temperature of the NaCl electrolyte according to the potentio-dynamic polarization measurements. The partly gold-coating extended the electrical conducting lifetime of the SCCY up to 690,000 s, despite the accelerated corrosion rates. The partly gold-coating, which is more cost-effective than full coating on the entire silver surface, is suggested as a practical approach to enhancing the electrical performance and durability of SCCY.

## Data Availability

The original contributions presented in the study are included in the article/Supplementary Material, further inquiries can be directed to the corresponding author.
